# Progress in Dentin-Derived Bone Graft Materials: A New Xenogeneic Dentin-Derived Material with Retained Organic Component Allows for Broader and Easier Application

**DOI:** 10.3390/cells13211806

**Published:** 2024-10-31

**Authors:** Lari Sapoznikov, Martin Humphrey

**Affiliations:** 1 Private Practice, Tel Aviv 6473925, Israel; lari.sapoznikov@gmail.com; 2 Private Consultant, 80802 Munich, Germany

**Keywords:** bone grafts, dentin, tooth, material, orthopedic, dental, xenogeneic, porcine, osteoinductive, ankylosis

## Abstract

The optimal repair of rigid mineralized tissues, such as bone, in cases of fracture, surgical resection, or prosthetic placement, is a complex process often necessitating the use of bone graft materials. Autogenous bone from the patient is generally the gold standard in terms of outcomes but also has disadvantages, which have resulted in extensive research in the field of tissue engineering to develop better and more convenient alternatives. In the dental field, several initiatives have demonstrated that the dentin material derived from extracted teeth produces excellent results in terms of repairing bone defects and supporting dental implants. Dentin is acellular and thus, in contrast to autogenous bone, cannot provide osteoblasts or other cellular elements to the grafted region, but it does contain growth and differentiation factors, and has other properties that make it an impressive material for bone repair. In this review, the beneficial properties of dentin and the ways it interacts with the host bone are described in the context of bone graft materials. Autogenous tooth material has limitations, particularly in terms of the need for tooth extraction and the limited amount available, which currently restrict its use to particular dental procedures. The development of a xenograft dentin-derived material, which retains the properties of autogenous dentin, is described. Such a material could potentially enable the use of dentin-derived material more widely, particularly in orthopedic indications where its properties may be advantageous.

## 1. Introduction

Bone defects may result from trauma, the surgical resection of tumors, degenerative diseases, congenital malformations, and dental surgeries or procedures [1,2,3]. For the correction of such defects, the natural healing process is often inadequate, and it is necessary to graft material into the defect to stimulate, enable, and direct the repair process while maintaining site stability and integrity. It is estimated that more than 2 million bone grafting procedures are performed annually worldwide, making bone the second most transplanted tissue after blood [1].

The gold standard graft material is autogenous bone from the same patient, because this not only provides a scaffold for host bone growth (osteoconduction), but also releases factors that stimulate host bone ingrowth (osteoinduction) and contains bone-producing cells that can locally generate bone tissue (osteogenesis). The harvesting of autogenous bone, however, requires extra surgical procedures and the properties of this material are not optimal for addressing all defects [1,3,4]. There has therefore been active development of alternative materials to improve outcomes and the ease of grafting in an economically viable way. In some countries, there is evidence of a trend of using autogenous grafts less often, combined with an increased use of allografts and biomaterials [5]. A great variety of materials have been examined, including bone tissue from other sources, natural biomaterials, synthetic materials, and different combinations of these materials (Table 1). Recently, there has been a focus on the development of complex tissue engineering materials with multiple tuned properties, that are often reactive to the tissue environment [6,7,8].

Despite the extensive literature on potential bone graft materials, only a limited number of materials have been introduced into the clinic [1,9,10]. This is due to the demanding requirements for a clinically effective material and also the costs associated with demonstrating that a material is really as safe and effective as anticipated before it can be introduced to the market.

A recent, promising and innovative approach in the dental field involves processing unwanted extracted teeth into an autogenous graft material, which is largely dentin derived, for filling extraction sockets and other bone defects, in order to restore functional dentition based on dental implants [11,12,13,14]. The success of this approach has shown that dentin combines several properties that are advantageous for bone defect repair (Table 1).

In this review, we examine the properties of dentin in the context of the theoretical requirements for an optimal bone graft material, compare it with existing materials, and make the case that dentin-derived material may have advantages for bone grafting beyond the dental procedures for which it has been developed. Autogenous dentin has even more restricted availability than autogenous bone, and to be developed for broader use there is a requirement for a dentin-derived material with less restricted availability. The development of a xenogeneic dentin-derived material that retains the key properties of autogenous dentin is described and the potential for further development and use in broader orthopedic indications elaborated.

## 2. Desirable Properties of an Ideal Bone Graft

### 2.1. Normal Bone Structure

Prior to defining the requirements for bone graft material, it is useful to examine the properties of the bone to be restored. Normal bone is a complex tissue that not only provides mechanical and structural support but is also important in calcium and phosphate storage, houses the bone marrow, and is also continually remodeling, effectively replacing itself [15,16]. Bone structure can be seen as a hierarchical construct at different dimensions, thus resulting in varying properties according to the requirements of the bone in question [17,18]. The structure within a single bone can vary considerably, presumably reflecting different local requirements in terms of tensional, compressive, bending, and torsional strength [19].

Bone is essentially a mineralized connective tissue consisting of approximately 40% organic material which is hydrated and provides flexibility and 60% inorganic material which provides rigidity and strength. The organic material consists largely of type I collagen and the inorganic material is crystalline biological hydroxyapatite [20,21]. At the nano-level, collagen is formed into multimolecular bundles which form fibrils that aggregate into larger bundles. The structural properties of bone at the nano-level are determined by the interaction of the mineral phase with the collagen. Hydroxyapatite crystals form in both intra- and extrafibrillary locations, thereby producing complex crystal aggregates that may have needle, platelet, or stacked platelet forms. The majority of underlying collagen is in well-ordered aligned arrays in which the mineral crystals are largely intrafibrillar and their orientation reflects the mechanical stresses that the bone is adapted to support [22,23]. This ordered material alternates with layers in which the collagen fibrils are disordered in terms of orientation, and where the mineral crystals are largely between the fibrils. The cellular components of bone such as the osteocytes are located within the disordered regions.

### 2.2. Normal Bone Growth and Remodeling

In the process of bone growth and remodeling, it is likely that the stress sensing of the osteocytes stimulates osteoblasts to produce ordered material in the orientation required to withstand the stress. During bone development and defect repair, the initial mineralization produces woven bone, in which the collagen fibrils and structure are disordered. A process of remodeling then results in the ordered layers of mature lamina bone which establish or restore the full resilience of the bone tissue [24].

### 2.3. Bone Repair and Optimal Graft Material Properties

From these considerations, it is evident that bone structure is both complex and dynamic. An ideal bone graft material should, as closely as possible, allow the re-establishment of the complex, mechanically optimized bone structure. This not only requires properties that simultaneously fulfill multiple parameters but also a change in properties over the period of healing that matches the natural responses of the host. Fortunately, bone is a naturally dynamic tissue continually adapting to the demands placed on it and so, generally, the elements for repair and restoration of the bone structure are present and just need to be supported and enhanced where possible [25,26,27].

Bone fracture or defect repair is characterized by several main phases of tissue response [24,27,28]. Initially, there is an inflammatory reaction in the blood clot filling the defect, during which neutrophils, macrophages, and other immune cells infiltrate, release cytokines and growth factors, start the removal of necrotic material, and stimulate the recruitment of wound healing cells. The subsequent ingrowth of capillaries, mononuclear cells, fibroblasts, and immune cells results in the formation of granulation tissue with the production of a collagenous extracellular matrix (ECM) which provides a substrate for the proliferation and differentiation of osteogenic cells, leading to the formation of a soft callus. As the ECM consolidates and osteoblasts are activated, mineralization of the disordered collagen fibers occurs, resulting in a more rigid hard callus composed of woven bone. This process is coordinated with ECM and vascular supply remodeling. Finally, there is an extended phase in which the woven bone is gradually remodeled into laminar bone which has similar function and strength to the original bone. There is considerable evidence that the intermediate soft- and hard-callus formation phases are sensitive to mechanical stress or stimulation, such that local stress or transmission of mechanical stress to the regenerating area can enhance the repair process [16,23,29] and there are advocates that bone fixation devices should allow for strain transmission at such critical phases [30].

Based on the above considerations of normal bone structure, turnover, and the phases of bone defect repair, the key characteristics of an ideal bone graft material can be listed as follows. An ideal graft material should:

1. bridge the defect with a structure that supports the growth of bone-generating cells such as mesenchymal stem cells (MSCs) and osteoblasts (osteoconductivity);
2. attract and sustain host cell ingrowth into the defect, particularly of bone producing cells (osteoinductivity);
3. create an environment that favors healing over inflammation;
4. not release any substances that are detrimental to healing or induce a tissue reaction (biocompatibility);
5. resorb slowly to retain defect/bone volume;
6. have mechanical properties that allow the transmission of forces in a range that encourages maintenance of host bone and remodeling of new regenerated bone as early as possible;
7. maintain defect site integrity during the remodeling to form mature bone;
8. eventually be resorbed or replaced by the host bone;
9. not interfere with host bone integration with prosthetic implants.

In addition to the properties required for optimal repair, there are several considerations relevant to the production, application, and approval of the material for clinical use, as follows:

1. adequate quantity of component/source materials readily available at a reasonable price;
2. readily controllable and economic production process;
3. consistent and reproducible properties and quality;
4. sterility and freedom from infection risk;
5. ease of storage and ready availability where required;
6. easy application to bone defect.

Although additional criteria may be possible, the aforementioned requirements cover the core essential criteria. As covered in many reviews of this area, no single material currently meets all these criteria, including those considered the gold standard [2,21,31].

## 3. Existing Bone Graft Materials

There are a wide range of bone graft materials that may be used for dental and orthopedic bone defect repair [1,2,3,9,10,21,31,32,33]. These include natural origin biomaterials such as autogenous bone, allogeneic bone, autogenous tooth, xenogeneic bone, and other xenogeneic tissues such as coral skeleton or seaweed minerals. Simple synthetic materials such as calcium phosphate ceramics or cements, bioactive glasses, or polymers have also been successfully used. Finally, there are composite materials with diverse components including growth factors (Table 1).

### 3.1. Autogenous Bone Grafts

There is consensus that the gold standard material is autogenous (also known as autologous) bone, because its structure and compatibility match the host tissue, and it is currently the only material that contains live bone precursor cells and is thus osteogenic, meaning that it contributes bone-making cells throughout the graft [1,4]. Clinical experience generally confirms that autogenous bone is superior to allogeneic and xenogeneic graft materials in most situations [4,33]. Obtaining autogenous material, however, requires a second surgical procedure on the patient and sometimes the amount of appropriate material is limited. Cancellous bone autografts contain the most osteogenic cells, although a variable proportion of cells undergo apoptosis during grafting, but they provide less structural support than cortical autograft material, which gives good support but has limited osteogenic cells and is largely osteoconductive. It is also possible to use bone marrow aspirate, which is more easily harvested and is osteogenic and osteoinductive, although the degree of osteogenicity is variable and it provides the least structural support. Thus, there is inherent variability in the properties of autogenous bone material which, when well-matched to the purpose, is not a problem. In some cases, however, cancellous material can resorb too quickly before new bone growth is complete, thus inadequately maintaining bone volume [1]. This is most evident for augmentation procedures in dentistry [32]. Despite the advantages of autogenous bone graft material, it is estimated that it is used in just 15% of grafting procedures in the US [3] and in some countries, the use is decreasing relative to substitute materials [5], showing that the limitations are significant.

### 3.2. Allogeneic Bone Grafts

Allogeneic bone material is harvested from human cadavers and treated to reduce the risk of infection or immune reaction [3,33]. It is available fresh, frozen, or freeze-dried. Most of the material comes from tissue banks which need to perform strict selection of cadavers in addition to sterilizing procedures to reduce potential infection risk. Structural allografts are used to treat large defects and for joint reconstruction, but these are not the focus of this review. Nonstructural particulate allograft material does not contain viable cells and so is not osteogenic but is potentially osteoinductive as it releases growth factors and provides structural support similar to autogenous material. Freeze-dried material is less osteoinductive than fresh/frozen material and has reduced mechanical strength [32]. Demineralization by acid incubation further weakens the mechanical strength but exposes the organic matrix, thus potentially releasing more growth factors [3]. Demineralized material is thus more osteoinductive but also less stable and more rapidly resorbed and so the balance of these aspects must be taken into consideration for particular uses. Although less effective than autogenous material, allogeneic material is used for many procedures. It obviates the need for additional harvesting from the patient but has more concerns regarding infection and has quite high batch variability.

### 3.3. Xenogeneic Bone Grafts

Xenogeneic bone graft materials are clinically used particularly in dental procedures and in countries where allografts are not readily available [3]. The most used material, for which there is also the most clinical evidence, is the deproteinized bovine bone material (DBBM) Bio-Oss. This consists of particles of bovine bone that have been progressively sintered at temperatures up to 300 °C followed by an alkaline treatment (NaOH), which results in the destruction of the organic component of the bone [34,35]. This processing eliminates any potential infectious or immunogenicity risk, but also eliminates the ECM and thus any organic osteoinductive factors. The particles are composed of bone-like hydroxyapatite, and they retain a surface structure resembling their bone origin, and they therefore provide a relatively natural physical substrate for ingrowth of the host bone. These particles have been particularly advantageous for bone augmentation procedures such as maxillary sinus augmentation or alveolar crest preparation for implant placement, because the material is very slowly resorbed, thus retaining the regenerated bone volume. Graft particles have been confirmed to persist even 7 years after sinus augmentation [36]. Similar materials have also been derived from porcine or equine origins [37].

It should be noted that in the category of DBBMs, there are also materials available that have been sintered at temperatures above 1000 °C [35,38]. At temperatures above approximately 400 °C, the crystal structure of the biological apatite changes from bone-like nanocrystallinity with incorporated carbonate to a highly crystalline form with low carbonate content. This influences both the mechanical quality (lower stability than bone) and solubility (lower than bone). A reduced release of calcium ions not only reduces the support of host bone growth, but it also decreases the resorption of the graft particles. Highly crystalline hydroxyapatite cannot be dissolved even at the low pHs generated by osteoclasts and macrophages and so the material becomes non-resorbable [3,39]. The reason for the very slow resorption of Bio-Oss is less clear. Biological hydroxyapatite resorption depends on multiple factors including particle size, the availability of osteoclasts, and the surface suitability for supporting cellular adhesion, in addition to the crystal and chemical nature of the material. Regardless of the detailed mechanisms involved, it is evident that the major advantage of such materials is that they maintain the regenerated bone volume but eventually remain trapped in the host bone, preventing formation of an optimal bone structure [40].

Apart from the slow- or non-resorbable xenogeneic bone graft materials, there are also bone-derived particulate materials that are less harshly treated and retain the endogenous organic matrix. Although there are potential concerns of immunogenicity and infectivity for such materials, the processing appears to be adequate and there has been no concern about these issues in clinical practice. In the dental area, such materials have been shown to be effective for the healing of bone defects and support of dental implants [41,42].

In the orthopedic area, the history of the attempted use of xenogeneic materials has been less successful and there are currently no xenogeneic graft materials approved by the FDA for orthopedic procedures. A review of orthopedic clinical studies using xenografting over the period 1966 to 2017 showed that, despite favorable results in 44% of the studies, 47% advised against the use of xenograft materials [43]. The studies variously used bone blocks, chips, and granules with different treatment protocols. The problems reported were high rates of graft non-union, failure of graft to integrate with the host tissue, and failure of the graft to remodel over time. In some cases, inflammatory responses necessitated removal of the graft material. The reasons for the failures are generally not well understood. One specific etiology is the exposure of alpha-Gal epitopes. Alpha-Gal is a cell membrane-localized carbohydrate expressed in most mammals except humans and old-world primates and against which an antibody response may occur. One study of ligament grafting has shown that even when an alpha-galactosidase enzyme pre-treatment reduced alpha-Gal in the graft by >99%, there was still an antibody response at two weeks after grafting, probably due to the lack of enzyme penetration to all membranes. In contrast, highly processed bovine bone grafts in which the organic material is denatured have been successfully used without any inflammation or immune reaction [44]. The most used xenogeneic graft used in dentistry, Bio-Oss, which has no organic content, has not been used in orthopedic procedures. The lack of resorption of such highly processed materials may also produce problems as there have been cases reported where particles have migrated or become encapsulated and act as a source of inflammation because they cannot be removed by the immune system [45]. This emphasizes the need to ensure that such materials are appropriately confined to ensure integration with the host bone.

### 3.4. Synthetic Alloplastic Grafts

Synthetic alloplastic materials are also available for bone repair. These largely comprise calcium phosphate ceramics or cements and a variety of, often complex, composite materials [31]. The basis for most synthetic graft materials are hydroxyapatites, which are structurally strong and resemble the mineral component of bone, and tricalcium phosphates, which are more readily resorbed but lack mechanical strength. This results in there being many different combinations of these two components being available. Composite materials generally combine such biomineral components with different polymers to create three-dimensional structures that facilitate bone regeneration. Simple synthetic materials are often available as pastes which are easily applied to irregular defects, while more complex structured materials can be prefabricated to fit the defect. Despite these properties, the synthetic graft materials to date are generally just osteoconductive and thus generally inferior to the tissue-derived materials [31]. Nevertheless, they are used in several indications for bone defect repair and may be used in combination with other materials. An active area of research is the combination of synthetic materials with growth factors and other active components to create osteoinductive graft materials.

### 3.5. Summary of Existing Graft Materials

None of the existing bone graft materials fulfill all the characteristics required of an ideal material. In particular, a material matching the required time course of events with respect to supporting and encouraging mineralized bone growth adequately, but then being resorbed at a rate that allows optimal remodeling and consolidation without compromising the mechanical properties, is lacking. Nevertheless, many are effective and set a basic standard for the development of improved bone graft materials.

## 4. Properties of Autogenous Dentin

It was appreciated at an early stage that dentin has rather special functional properties in relation to the interaction with bone. Early studies demonstrated two key phenomena highlighting these special properties, namely bone–dentin integration in tooth ankylosis and the ability of dentin to induce bone formation in non-skeletal tissues.

### 4.1. Ankylosis and Replacement Resorption

Normally, dentin and bone do not come in direct contact because a connective tissue layer known as the periodontal ligament separates the tooth root from the alveolar bone. In cases of trauma, the periodontal ligament may degenerate, thus allowing the root dentin to come into direct contact with the alveolar crest bone. In such cases, the alveolar bone was not only maintained, rather than regressing as is usual following tooth removal, it also formed a direct mechanically robust contact with the dentin, and over time the tooth dentin was slowly replaced by bone in the process of normal bone turnover [46,47,48]. This phenomenon of ankylosis with external replacement resorption showed that dentin has a structure that readily integrates with bone such that it is incorporated into the normal turnover of bone with no inflammatory reaction and no loss of mechanical integrity [49,50].

This slow replacement of the tooth root by bone is so robust that the best permanent treatment option for ankylosis, introduced by Malmgren and colleagues in the 1980s, is to remove the exposed enamel-coated part of the tooth (decoronate) and insert a dental implant into the site of the tooth root [46]. The slow replacement of the dentin by bone ensures a robust integration of the implant while maintaining adequate mechanical support. The integration of dentin with bone works well because the mechanical strength is similar to that of cortical or compact bone (Table 2) [51,52,53,54,55,56,57,58].

The precise and reproducible measurement of the mechanical properties of mineralized tissues is challenging and depends on the methods used and the treatment of the tissue and therefore, the ranges of values are most representative. Nevertheless, it is evident that the elasticity and the compressive and tensile strengths of dentin and cortical bone are very similar while the bulk properties of trabecular bone are much weaker (Table 2), although the mechanical strength of individual trabeculae is closer to that of bulk cortical bone [59]. As would be expected, the mechanical strength of dentin is largely determined by the mineral component with modification by the collagen matrix, so that the mechanical strength of demineralized dentin is much lower than that of native dentin [51,58,60].

### 4.2. Osteoinductivity

The second early appreciated property of dentin is that its demineralized matrix is osteoinductive, in the sense that when implanted in non-bone tissue it induces the formation of bone by attracting host mesenchymal cells and promoting their differentiation to the osteoblast pathway [13,61,62]. Thus, although dentin is produced by odontoblasts, is acellular, and has a different structure and composition to bone, it induces bone formation rather than dentin formation when implanted in other tissues. It is only when dentin particles are implanted into tooth cavities or the pulp chamber that odontoblast, rather than osteoblast, ingrowth occurs, resulting in dentin regeneration [63]. This property of the dentin ECM potentially offers clear advantages for bone regenerative applications.

Multiple properties of dentin may contribute to its osteoinductivity, promotion of mineralization, and formation of bone tissue. The pioneering work of Urist and colleagues showed that diffusible factors were partly responsible for the stimulation and differentiation of osteoblasts, leading to the identification of bone morphogenic proteins (BMPs) [64,65,66]. Subsequently, characterization of the organic matrices for dentin and bone has shown that while both are largely composed of type I and other collagens, they also contain a wide range of non-collagen matrix molecules, growth factors, neuropeptides and plasma proteins [63,67,68].

There is overwhelming evidence that the extracellular matrices of dentin and bone are bioactive on multiple levels in terms of promoting mineralized tissue production [63,69,70,71,72,73]. Dentin ECM contains and releases signaling molecules such as BMPs [74,75], TGF-β [76,77,78], VEGF [79,80], PDGF [79], FGF-2 [79], PIGF [79], and IGF [81]. This bioactivity has led to initiatives for developing dentin ECM as a scaffold for bioengineering approaches for repairing damaged tissue [82]. It should be noted that these growth factors are stabilized and preserved within the mineralized matrix to the extent that they can still be extracted and identified from archeological specimens several hundred years old [83].

In addition, the dentin ECM contains several non-collagenous proteins that have been identified to play critical roles in the differentiation and production of mineralized tissue. These include the small integrin-binding ligand N-linked glycoprotein (SIBLING) family, all of which display an Arg-Gly-Asp (RGD) motif that mediates cell attachment/signaling by binding to cell surface integrins [84]. Members of this family include osteopontin, bone sialoprotein (BSP), dentin matrix protein 1 (DMP1), dentin sialophosphoprotein (DSPP, proteolytically cleaved to dentin sialoprotein, DSP, dentin phosphoprotein, DP, and dentin glycoprotein, DGP), and matrix extracellular phospho-glycoprotein (MEPE). These molecules are intimately associated with mineralized tissue and can promote or inhibit the nucleation of hydroxyapatite (HA) and in some cases also stimulate osteoblastic differentiation of MSCs (DMP1, DPP). Other bioactive non-collagenous proteins include dentin matrix proteins (DMPs), osteocalcin, and bone sialoprotein. Some of these bioactive proteins are readily released such that simple incubation in fluid or acid demineralization results in osteogenically active molecules being released, while other active molecules are more tightly bound to the ECM [85]. TGF-β, DSP-1, FGF, and VEGF, for example, are more readily released [78,86].

The therapeutic application of individual growth factors such as BMP-2 and BMP-7 has problems where the supraphysiological levels required may lead to ectopic bone formation and inflammation. In contrast, the dentin ECM has been termed a physiologically optimized matrix for stimulating osteogenic signaling, as it releases a combination of growth and differentiating factors at levels that promote both the ingrowth and differentiation of MSCs to form osteoblasts [71].

The inorganic component of dentin probably also plays a role in inducing an osteogenic environment that promotes bone repair. It is well established that both calcium and phosphate not only contribute to mineralization by providing the components for hydroxyapatite formation but are also key in signaling the differentiation and proliferation of osteogenic cells [87]. Extracts of dentin from which the organic component has been eliminated by heat treatment have been shown to be active in promoting the osteogenic differentiation of bone marrow-derived MSCs in vitro [88]. Furthermore, in bioengineering approaches, it has been shown that by placing small completely demineralized dentin particles (<40 μm) in a hydrogel matrix that contains amorphous calcium phosphates, the material attracts MSCs and results in good osteogenesis and mineralization, demonstrating the positive interaction between the inorganic and organic components [89].

Apart from the obvious bioactive molecules in dentin ECM that contribute directly to osteoinductivity and local mineralization, there are other molecules that are important. Matrix metalloproteinases (MMPs) are ECM-located degradative enzymes which are important for wound healing, and act via both the degradation of collagen or collagen fragments and by activation of growth factors that are bound to the matrix or secreted in an inactive form [90,91,92,93,94]. The MMPs found in dentin include MMP-2, -3, -7, -8, -9, -13, -14, -20, -23, and 25 [91]. MMP-8 is the most abundant [95] and is retained and still active even after processing procedures such as demineralization [96]. MMP-8 appears to have largely degradative activity and is associated with caries development [95]. Treatment of demineralized dentin matrices with recombinant MMPs demonstrated that the released molecules have dentinogenic, osteogenic, and angiogenic effects [92].

### 4.3. Preparation and Clinical Use of Autogenous Dentin as a Bone Graft

A major driver for the development of tooth dentin as a bone graft material was that in many of the dental procedures where bone defect grafting is required, teeth are extracted and simply discarded as waste. Development of a safe and adequate processing procedure to produce particulate or block dentin from a patient’s own teeth has clear advantages and being autogenous, has no concern for potential immune or infection issues. The processing should produce a dentin material retaining the key bone growth supporting properties while ensuring the absence of contamination by infectious agents.

**Figure 1:**
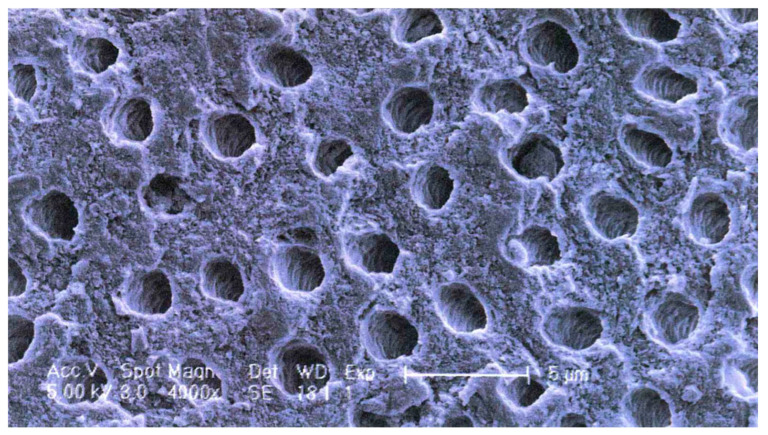
Dentin microstructure: SEM showing tubular arrangement. White scale bar = 5 μm.

Apart from the special functional properties of ankylosis/replacement resorption and osteoinductivity, dentin as a scaffold material for new bone growth (osteoconductivity) differs from bone, particularly cancellous bone. As reflected in the mechanical properties indicated in Section 4.1 (Table 2), the dentin collagen/hydroxyapatite structure is denser, a property which may contribute to the slower resorption. Instead of having a trabecular or compact structure, dentin contains a relatively regular array of tubules in which the odontoblast processes run (Figure 1). This microstructure is an ideal surface for osteoblasts and osteoclasts to adhere to and penetrate.

**Figure 2:**
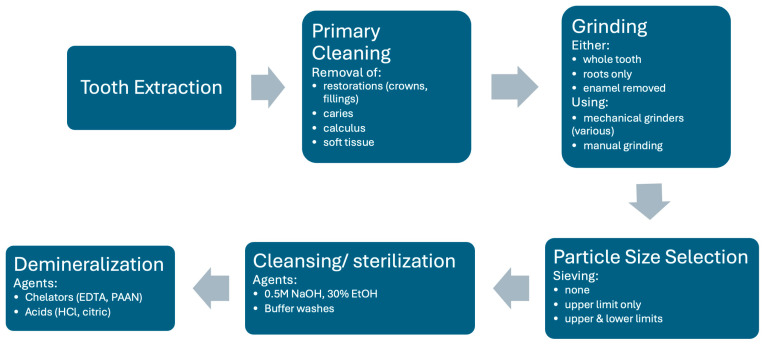
Generic flow diagram for autogenous dentin production to illustrate key possible processes. Note that individual methods may omit certain steps or perform them in a slightly different order. Listed aspects in each step are the range of alternative methods. See text for details.

Processing of the tooth should therefore retain and open the tubules while keeping the extracellular matrix with its growth factors as intact as possible and retain sufficient mechanical strength in either the ECM or mineral structure. The extracted tooth can either be processed into particulate material or into blocks, which are slices with the dimensions restricted by the tooth size. Particulate material is largely used due to its flexibility, although there must be procedures to ensure its stability in the defect. The generic processing procedure is outlined in Figure 2.

Following extraction, the tooth is generally washed, soft tissue (pulp, dental ligament) removed, any restorations such as crowns or fillings removed, as well as any calculus or caries, using a high-speed dental bur. From this stage, there are many different procedures described in the literature resulting from parallel development by different centers.

The main required component is dentin. Some procedures attempt to produce pure dentin material by either coronating and just using the roots, or by removing the enamel layer with a dental bur. Other procedures use the whole tooth so that enamel particles are also present.

The tooth is then ground into particles. In some cases, manual grinding has been used. In recent times, electric mechanical grinding machines that provide greater consistency have become more common. Different machines have been used, some with rotating blades and others with a more crushing mechanism. This process produces irregularly shaped particles in a wide size range. The aim is to produce particles or granules that are greater than 200–300 μm in diameter, as smaller particles are more rapidly resorbed and leave smaller gaps for the ingrowth of granulation tissue. Upper size limits generally vary between 800 and 1200 μm, although for space filling procedures particles up to 2 mm may be used. Most machines use screens with different sized grids to filter out undesirable particles that are too small and too large. In some cases, however, only exclusion of larger particles is performed.

The grinding of fresh teeth with the complete organic component tends to create a smear coating of organic material on the surface of the particles. To remove this coating and expose the tubules, a washing step is performed. Most commonly for mineralized particles, this involves incubation in an ethanolic alkaline solution (0.5 NaOH, 30% EtOH) followed by phosphate buffer washes to remove the cleaning agent and restore the pH. This results in a surface appearance as shown in Figure 1. This solution also sterilizes the material. Note that such solutions do not demineralize the dentin. Such washed fully mineralized dentin material (MDM) particles can be directly implanted into a bone defect. Due to the size and packing of the particles, bone defects of approximately twice the volume of the original tooth can be filled.

Many groups find it desirable to remove some or all of the mineral component of the dentin material because it has been shown in vitro that demineralization releases osteogenic growth factors. This procedure, however, reduces the mechanical strength of the material, and it is not clear whether growth factor release is enhanced in vivo because the factors can be washed out in the processing and degradative enzymes are also released that may degrade them (see later sections for a more detailed discussion on the consequences of this processing). There is a great variety of demineralization procedures described in the literature, although all materials are generically described as demineralized dentin material (DDM). Different agents can be used to dissolve the hydroxyapatite component of the material. The use of chelating agents such as EDTA which bind multivalent positive ions such as calcium and magnesium is a common method. Recently, higher molecular weight chelators such as sodium polyacrylic acid (PAAN) have also been used. Acid treatment is also effective for demineralization, most commonly hydrochloric acid. Different incubation times and concentrations of these agents have been used. There is evidence that the demineralizing agents may alter the ECM structure and long incubations may wash-out growth factors, but nevertheless there is good evidence for the efficacy of all published varieties. In some cases, virtually complete demineralization is achieved and, in others, only partial demineralization. Some centers do a short EDTA treatment of mineralized particles to achieve a surface demineralization of maximally the first 20 μm only. Some commercial devices perform automatic demineralization following particle preparation, and it is not clear which agents are used for what durations. In cases where the material is processed in a central facility for later use, additional steps may be added to sterilize and preserve the material. This includes drying or lyophilization, sterilization treatment (irradiation or gas), or heat treatment.

There is a clear lack of standardization of processing procedures, which complicates the clinical evidence-based selection of critical process steps. Nonetheless, there is evidence of clinical efficacy for a wide range of processed dentin-derived graft material, as outlined in more detail in the next section.

Clinical application of the particulate dentin material follows the general procedures for the use of particulate bone. The aim is to have a complete filling of the defect with intimate contact between the walls of the defect and the material without having the material packed too tightly as this can inhibit tissue ingrowth. The material can be premixed with blood from the defect or just inserted. For augmentation procedures where the bone is intact, the surface should be prepared by drilling to provide access to the vasculature or marrow. The material should be stabilized and where an enclosing wall is absent a membrane should be placed. Even where there is stability, the use of a membrane can be beneficial to prevent rapid soft tissue ingrowth. The material can also be mixed with other materials when desired. Commonly, platelet-rich fibrin (PRF) has been used, which helps with filling and stabilization of the particles, as well as providing extra growth factors. When there is insufficient material, dentin particles have been mixed with either autogenous bone or DBBM.

### 4.4. In Vivo and Clinical Evidence for Dentin Efficacy as a Bone Graft

Based on the above properties, and that extracted teeth have just been considered as waste, several groups have developed approaches to use the patient’s own extracted teeth as a source of material for bone grafting in dental procedures.

One approach is to send the teeth to a central facility specially set up for processing, which has the advantage of having a strict control over the processing steps but makes the procedure rather complicated and expensive [97]. This is generally performed on an individual patient basis, but there are initiatives to develop a process suitable for allografting [98] and there are limited clinical data supporting this [99].

An alternative is chair-side processing using special grinding machines and prepared chemicals for the production and sterilization of the material [11,14,100,101]. This allows for use of the material in a single session immediately following the tooth extractions. In particular, due to the ease of the latter process, the use of autogenous dentin as a bone graft material is becoming more popular, despite restrictions such as the condition of the teeth and whether there is sufficient material available.

There is therefore a growing body of evidence on the clinical efficacy of tooth-derived, dentin-derived materials in a range of dental procedures involving bone defect repair. To review the status of the use of dentin-derived bone graft material, a PubMed search was performed on 28 September 2024. PubMed was used as it is extensive but uses criteria to ensure a degree of scientific quality and integrity. The search algorithm used was ‘dentin AND (bone AND graft AND material)’ in all fields. A filter restricting hits to the last 5 years resulted in 112 publications being identified.

The publications were classified according to multiple parameters (assessable/relevant, article type, study type, material type, preparation method, particulate or block—spreadsheet available on request). This resulted initially in the exclusion of 24 articles as not being relevant or informative for the aim of the search (14 not relevant to dentin as graft material, 4 not in English or German, 3 in non-relevant indications, 1 correction notice, 1 quality assessment of another article). Thus, 89 relevant articles were examined in detail. A total of 19 articles were reviews without new data, while 70 were original works with new data. Most articles focused on autogenous dentin (79 overall, 15 reviews, and 64 original articles), with just six examining allogeneic dentin and three xenogeneic dentin materials, and one review covering both autogenous and allogenic materials. All clinical data were derived from use in dental indications. Some animal studies, however, showed good bone repair in tibial models [102,103].

To examine the overall clinical efficacy of autogenous dentin-derived material in dental indications, both the reviews of clinical studies (16 publications, Table 3) and original clinical studies (46 publications) were examined. Seventeen of the forty-six original clinical studies were comparative (Table 4) with the remainder examining dentin use without controls.

The review articles all showed evidence that autogenous tooth-derived dentin materials were effective for bone repair in a variety of dental procedures (Table 3). Most reviews cautioned that many studies had relatively low patient numbers and that there was a general lack of long-term data, and therefore, larger studies with longer follow-up would be desirable. Within these restrictions, however, it was evident that in many cases dentin-derived graft materials were at least as good as the well-established autogenous bone and xenogeneic DBBM. The properties leading to this good clinical performance are a combination of osteoinductivity leading to good new bone growth, and a resorption rate that is slower than autogenous bone material but not as slow as the deproteinized xenogeneic material (DBBM, Bio-Oss), which in many cases appears to be effectively non-resorbable. The slow resorption combined with intimate contact with new bone underlies stable volume maintenance that is particularly important in sensitive bone regions such as the alveolar ridge, which rapidly resorbs after tooth extraction. In such cases, the dentin-derived material appears to be superior to autogenous bone, which resorbs too quickly, and offers similar volume maintenance as DBBM but without being non-resorbable.

Over the last five years, we located 46 original clinical studies that have further confirmed the conclusions of the review articles (Table 4). Alveolar ridge preservation was the most common indication, as this is most challenging in terms of preserving bone and is a procedure that provides extracted teeth for the procedure. Many different methods were used, which made comparisons difficult. This partially reflects the differing approaches in different centers, but also the combination with other components thought to be beneficial for bone and/or soft tissue healing. Combination with PRF, for example, was commonly used. Other aspects such as the use of membranes clearly differed between studies, although such aspects were not always reported. In terms of the material processing, 16 studies used demineralized dentin while 25 used mineralized dentin and 4 studies used both in a comparison (see below for more detail on this aspect). Although non-comparative studies are useful to show that a material can effectively be used in a certain indication, comparative studies are necessary to clearly establish how the material compares to other commonly used materials or procedures. The comparative studies have therefore been summarized in Table 4.

The first six studies in the table are well-controlled studies comparing dentin-derived material with natural or blood clot healing. The first three show that the dentin material is clearly superior to natural healing for ridge augmentation and stabilization of the second molar after removal of an impacted third molar which leaves a large unstable bone defect [118,119,120]. The second three show similar superior performance over natural healing for ridge preservation after tooth extraction [121,122,123]. This provides solid evidence for an added value beyond natural healing in these indications.

One study showed that dentin-derived material mixed with PRF was superior to PRF alone for ridge preservation [124]. Although an advanced PRF was used that contained numerous growth factors, this was not adequate to maintain the ridge dimensions, whereas the dentin material prevented bone loss up to 4 months, as assessed both clinically and radiographically.

Three comparative studies showed equivalent or superior bone repair with dentin material in comparison to autogenous bone in situations where block material with particulate was used for bone augmentation [125,126,127]. Apart from good bone growth and integration with the dentin, the slower resorption of dentin compared to autogenous bone was beneficial in these procedures.

Another study demonstrated superior performance for ridge preservation for dentin material combined with PRF over allograft material combined with PRF, despite the latter being considered as an adequate grafting method [128].

A comparison with xenogeneic de-proteinated bovine bone material (DBBM) has been performed in four studies. Three studies used Bio-Oss which is poorly resorbed [130,131,132] and one used Cerabone, which is high-temperature sintered and non-resorbable [129]. DBBM is widely used where volume maintenance is critical because very slow or non-existent resorption provides a solid structure during bone ingrowth but then complicates remodeling. The dentin-derived material had a similar good ridge preservation/augmentation with a higher proportion of new bone than either DBBM alone [130] or a mixture of DBBM with autogenous bone [129]. Another study showed that when grafting is performed with immediate implant placement, the dentin-derived material showed less stability than DBBM early after implant loading, although stability after 12 months was similar. The earlier instability may be due to more remodeling occurring with dentin than the poorly resorbable DBBM. It may be anticipated that the higher proportion of bone may be advantageous at longer follow-up. When used for ridge augmentation to enable orthodontic tooth movement, dentin provided a greater early augmentation than DBBM with milder post-operative side effects but in the longer term had a similar ridge volume due to remodeling of the graft site [132]. Finally, it has been shown that dentin can be mixed with DBBM to extend its use, but that up to 4 months, there is no difference in site preservation but less new bone growth due to the space taken up by the poorly resorbed DBBM [133]. It can be concluded from these comparative studies that dentin-derived material provides similar or greater volume retention or augmentation to DBBM and, in general, undergoes slow resorption to allow for greater bone growth in the grafted site, whereas DBBM is hardly resorbed.

Dentin-derived material was superior to β-tricalcium phosphate (TCP) for site repair and stabilization after third molar coronectomy [134]. TCP is a synthetic bone graft material known to be effective for promoting bone repair which has a relatively rapid resorption. The promotion of new bone growth and site stability provided by the more slowly resorbed dentin more effectively prevented movement and rotation of the retained root of the third molar and maintained sufficient support to stabilize the second molar adjacent to the defect.

Thus, although the use of dentin-derived materials is a relatively new technique, there is already considerable evidence supporting their use for both repair and maintenance of bone defects and the augmentation of bone where required. Despite the desire for larger controlled clinical studies with longer follow-up periods, as outlined above, there is a consensus among the recent reviews that dentin-derived material can be as effective, and sometimes better, than the standard materials such as autogenous bone or DBBM. In many cases, good quality comparative studies have demonstrated the relation of dentin-derived materials with other materials.

Different approaches to processing dentin have been used. The simplest method is to use fully mineralized tooth or dentin particles which are ground to a size range of ca. 300–1200 μm and treated with a cleansing and sterilizing solution [11,135]. Such material provides mechanically strong particles that can interact with the ingrowing host bone. This method has been shown to be effective for repair and implant placement in dental procedures [11,100,108,118,119,120,121,129,130,135,136,137,138,139,140], as have non-demineralized dentin blocks [107,126]. It has been shown that non-demineralized dentin released more growth factors such as BMP-2 than autogenous bone in vitro [141], thus stimulating host osteogenic activity. The regenerated site is relatively quickly (3–4 months) strong enough to accept a dental implant. The crucial parameter for mineralized particles is that they are > 200 μm in size as smaller particles are more rapidly degraded and have less inter-particular space for cellular and blood vessel ingrowth [142,143,144]. It has been shown that the particles do not interfere with the firm contact between the host bone and dental implants [138,145]. Such particles have also been shown to generate new bone at sites remote from the edges of the defect, confirming an osteoinductive effect [139]. The grafted areas demonstrated good host bone ingrowth and a superior density of host bone to graft material contacts compared to bone material [135], thus allowing for extensive ankylosis-type contacts.

An alternative method is to process the dentin or tooth particles with a demineralizing solution. Demineralization has been shown in vitro to release many growth factors and non-collagenous active molecules that theoretically could generate an osteostimulatory environment immediately after implantation [72]. In practice, however, full demineralization leaves the ECM vulnerable to both endogenous degradative enzymes, such as MMPs, and also to the action of inflammatory cells, and may wash out some factors. So the degree to which these factors play a sustained role in vivo is debatable. Nevertheless, fully or partially demineralized dentin has also been shown to be clinically effective as a bone graft material for dental procedures [105,106,109,113,122,145,146,147,148,149]. Some in vivo studies have shown that partially demineralized material may be more effective because it potentially produces some elevated early growth factor release but also a sustained release of factors as the mineral phase is slowly degraded [101,143]. The retention of the mineral component also provides a mechanically stronger graft.

There is no standardized protocol for demineralization and so there are likely differences between the materials described as demineralized, both in the extent of demineralization and the composition of the remaining ECM. Demineralization can be achieved either with acid treatment (for example, HCl or nitric acid) or with calcium chelating agents such as EDTA [113,149]. An interesting recent approach is to use high molecular weight chelating agents which can penetrate into the tissue but not into the collagen fibrils, thus removing the interfibrillar mineral but not the intrafibrillar mineral [150]. The claimed advantage is to maintain a better mechanical strength while releasing more osteostimulatory molecules. For loosely bound growth factors and non-collagenous proteins, there appears to be no difference between acid and chelator demineralization. It is, however, unclear to which extent the factors are retained or washed out with different protocols. Influences on factors such as MMP enzymes may also be different. There is therefore a need for more study and standardization of demineralization techniques, particularly in consideration that the non-demineralized material is effective.

There is some limited clinical experience showing that the dentin-derived material can be combined with other materials such as deproteinized bovine bone [133,151] and autogenous bone [152].

In summary, although there is an overall need for studies with larger numbers of patients and longer follow-up periods, there is already relatively strong evidence that dentin-derived bone graft material is indeed an effective and potentially superior bone graft material for dental procedures. Both mineralized, partially demineralized, and fully demineralized dentin materials have been shown to be effective, although there may be subtle differences between the materials. Certainly, a direct comparison of mineralized with partially demineralized material did not show major differences with early clinical follow-up [153], nor in animal models [154]. The standardization of processing methods and careful comparison of different types of dentin-derived graft material will be required to ascertain the most appropriate material for each use.

### 4.5. Current Limitations of Autogenous Dentin Material

As for all graft materials, there are potential and actual limitations for autogenous dentin material. The method requires that teeth are extracted to obtain the material, which may not be the case particularly for the augmentation of bone or repair of defects such as cysts. The amount of material obtained is also limited, making it less suitable for major reconstructions, although it may be mixed with other materials. Mixing with other materials, however, mitigates some of the advantages of the material. Some studies have used tooth/dentin slices for block purposes, but the tooth dimensions limit the region that can be covered. The material is therefore largely limited to particulate processing.

The current evidence for the efficacy and safety of autogenous dentin material for dental procedures is dominated by studies with relatively short-term follow-up. Individual cases have shown stability for several years but larger studies with longer follow-up would be desirable. Currently, there are many different processing procedures being used for the preparation of the material. Although acceptable results appear to be obtained despite this situation, it would be desirable to have more clinical evidence-based justification of particular methods. For mineralized dentin, the variation is moderate but with demineralized materials, the range of processing methods is considerable. Several proprietary materials also do not clearly disclose the details of the processing. This situation is understandable when a new interesting approach is becoming established and there is a desire to try out many different methods and also combinations with other procedures to achieve optimal performance. Nevertheless, more solid studies would increase confidence for the methods to be used more widely with appropriate adjustments according to the individual situation.

## 5. Theoretical Optimal Use of Autogenous Dentin

**Figure 3:**
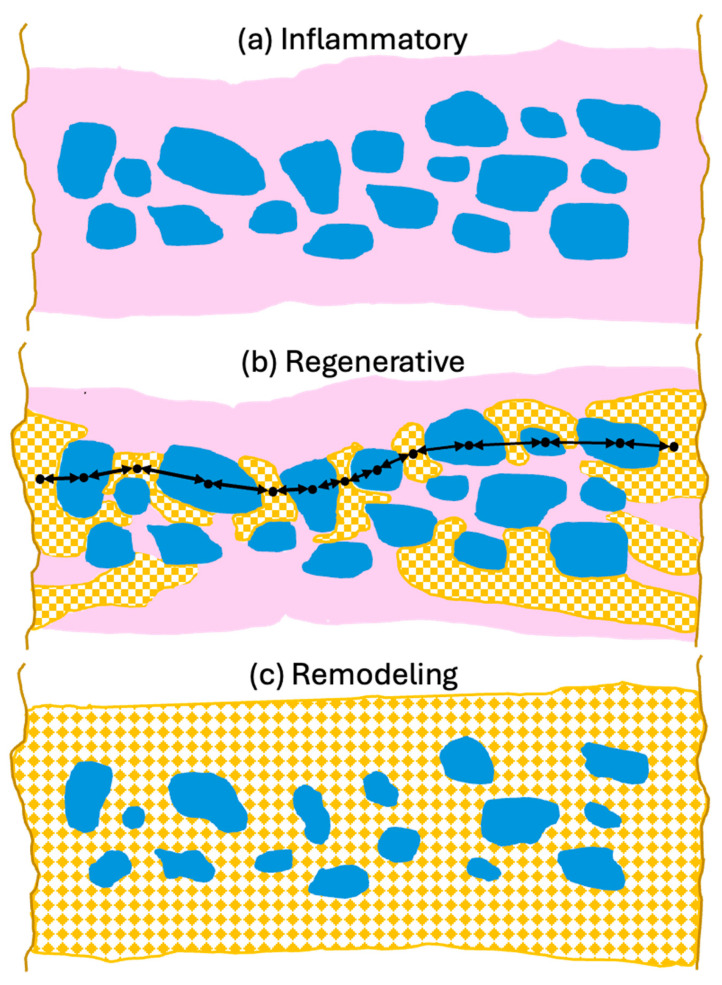
Schematic overview of dentin properties in each phase of bone defect healing. (**a**) Inflammatory phase: Macroscopically, dentin particles (blue) are surrounded by a blood clot (pink) which is infiltrated by inflammatory cells, fibroblasts and MSCs. Microscopically, the dentin releases factors which attract neutrophils encouraging removal of debris and factors which promote differentiation of neutrophils and macrophages to the anti-inflammatory, wound healing N2 and M2 phenotypes. Dentin attracts MSCs and induces osteogenic differentiation initiating woven bone production. The dentin also releases angiogenic factors which encourage vessel growth into the grafted area. (**b**) Regenerative phase: Macroscopically woven bone (patterned orange) forms near the defect edges and the dentin particles to form mechanically robust bridges (black arrows) where bone ankyloses with the dentin. Microscopically, the dentin particle surface and tubules allow for intimate contact of osteoblasts with the graft particles. The osteoblasts accept the mineralized dentin as bone hydroxyapatite and form more mineralized tissue. Transmission of mechanical force stimulates mineralization. Non-inflammatory external replacement resorption via bone remodeling units is initiated. (**c**) Remodeling phase: The dentin particles are integrated into the bone structure which evolves to laminar bone (patterned orange). The similar mechanical strength of the dentin means it is well matched to the bone thus reducing stress fracturing. Remodeling of the bone results in a slow non-inflammatory resorption of the dentin particles while maintaining mechanical strength. Diagram is schematic to illustrate principles and not to scale.

To support optimal bone repair, a bone graft material must change appropriately over time to match the requirements of the ingrowing host bone. To highlight the advantageous properties of dentin and how they may best contribute to bone repair, it is useful to describe them in the context of the bone healing response as described in Section 2 and illustrated in Figure 3.

### 5.1. Initial Inflammatory Phase

The initial phase in bone defect repair is an inflammatory reaction to the traumatic or debridement trauma which then optimally rapidly evolves into a wound healing phase [24,27,28]. The critical role of the immune system in the success of bone repair, particularly in the context of biomaterial-enhanced approaches, has been increasingly recognized recently [155]. The initial reaction is important in removing damaged cells and material and attracting increased blood flow and the necessary cells to establish granulation tissue for repair to begin.

The dominant cell type in early phases are the neutrophils, which continually monitor tissues for pathogen-associated molecular patterns (PAMPs) and enter bone fracture hematomas within minutes of injury [156]. These are followed by monocytes, macrophages, and MSCs. The early reacting innate immune cells can exhibit either pro-inflammatory or anti-inflammatory and growth-promoting phenotypes. Current evidence suggests that an active but not excessive inflammatory phase is beneficial for bone healing if it transitions to an anti-inflammatory and pro-healing phase [157]. The conditions in the bone defect determine the extent of the inflammatory response and how rapidly it transforms into a healing situation and a component of this is the introduced bone graft material.

In this first phase, the grafted bone graft material particles fill the defect and are surrounded by the hematoma and tissue exudate, through which the invading blood and local tissue-derived cells migrate (Figure 3a). Dentin-derived materials are known to have good biocompatibility, and they do not promote a large inflammatory response when implanted. Furthermore, there is evidence that dentin-derived factors may act to promote an anti-inflammatory and growth-promoting environment. It has been shown, for example, that a crude extract of demineralized dentin attracts neutrophils and that this effect is mimicked by the dentin-specific non-collagenous extracellular matrix proteins, dentin phosphoprotein and dentin sialoprotein [158]. Thus, release of these factors from dentin may help to recruit neutrophils and establish a robust initial inflammatory neutrophil response. Recent work has suggested that such a robust response not only promotes the removal of damaged material but also can enhance bone MSC recruitment for the healing phase by the generation of anti-inflammatory N2 neutrophils [159,160].

Dentin extracts have similarly been shown to promote the polarization of macrophages towards the anti-inflammatory, wound healing M2 phenotype rather than the M1 inflammatory type [161], thus inhibiting the inflammatory reaction and promoting healing. The timely switching of macrophages from the M1 to M2 phenotype is crucial for immunosuppression, resolution of inflammation and osteogenesis [162]. The same study also showed that the extracts inhibited osteoclastogenic activity, which would inhibit bone resorption and possibly the immediate resorption of the grafted particles [161].

Interestingly, there is clinical evidence that this anti-inflammatory influence may even affect adjacent tissues. For example, gingival soft tissue inflammation was reduced and healing improved with dentin-derived material when used for alveolar ridge preservation [122].

It should be noted that an initial phase of inflammatory reaction is probably beneficial for longer term healing, as it removes damaged tissue and promotes infiltration by appropriate cells, but this phase should only be transient and followed by a healing reaction. The likely critical phase for a positive dentin influence is therefore after the initial inflammatory reaction, suggesting that mineralized graft material may be superior as it releases factors over an extended period as the mineral component is gradually degraded by the natural resorption process. Having an enhanced release of factors very early, as promoted by demineralization, may therefore have a limited impact. This is supported by the finding that the impregnation of collagen membranes with an acid extract of dentin ECM did not influence bone repair in a rat calvaria-defect model [163]. Thus, by slowly releasing factors that influence immune cell polarization, dentin suppresses the immune reaction and inflammation and encourages a healing response with osteogenesis.

### 5.2. Regeneration Phase with Osteogenic Cell Infiltration, Woven Bone Formation, and Vascularization

As the inflammatory response resolves, the dominant processes involve the ingrowth of vasculature and the formation of granulation tissue that forms the matrix for woven bone formation. As summarized in Section 4.2, dentin particles release both organic and inorganic molecules that attract osteogenic precursor cells and promote their differentiation and similarly promote angiogenesis.

**Figure 4:**
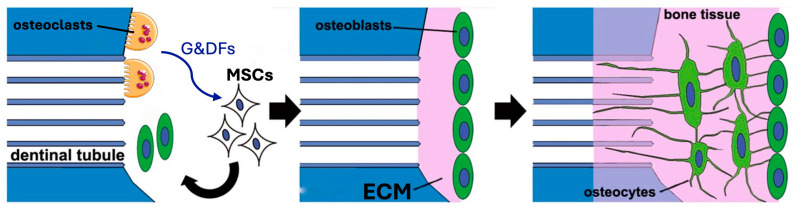
Bone production on the surface of mineralized dentin particles. **Left:** Osteoclasts release growth and differentiation factors (G&DFs) as they resorb the dentin. These factors attract MSCs and induce their differentiation to osteoblasts. **Middle:** The osteoblasts generate new ECM which mineralizes. **Right:** As the bone tissue extends and matures, the osteoblasts differentiate to osteocytes establishing normal bone structure. At later stages, osteoclast/osteoblast bone remodeling units form which slowly replace the dentin. Modified from Tanoue et al. 2018 [164] using Servier Medical Art, licensed under a Creative Commons Attribution 4.0 international license (https://creativecommons.org/licenses/by/4.0/).

On the macroscopic scale, however, the critical aspect is the formation of intimate ankylosis-like contacts between the forming woven bone and the dentin graft particles that allow for the establishment of mechanically robust networks of bone and graft (Figure 3b). Such contact is encouraged not only by the release of factors from the dentin particles but also by the microscopic surface structure characterized by tubular pits. It has been shown that osteocytic precursors and osteocytes readily grow on dentin surfaces [70,154,164] where they upregulate osteogenic markers. A comparison of mineralized and partially demineralized graft particles did not show a major difference in cellular activation [154]. Comparison of the osteogenic differentiation between dentin and DBBM showed that dentin was superior [70]. In vitro studies have also shown that dentin provides an excellent substrate for the adhesion of osteoclasts with an 11-fold higher resorption than for bone [165], although once adhesion occurs the actual resorption activity appears similar and is also not dependent on the orientation of the collagen at the surface. As summarized in Figure 4, we therefore believe that, at the microscopic level, factors released from the dentin attract and induce differentiation of MSCs to form osteoblasts on the surface of the dentin, thus resulting in bone generation in intimate contact with the dentin surface. At later stages, osteoclast/osteoblast bone remodeling units slowly resorb the dentin, replacing it with bone.

Formation of a mechanically robust bone-graft network based on ankylosis, and the similar mechanical properties of dentin and compact bone (Table 2), would be expected to promote excellent functional bone regeneration because the transmission of mechanical stress positively influences the appropriate turnover of the bone structure [16,23,29]. Interestingly, the dentin particles do not seem to interfere with the close apposition of the host bone with titanium dental implants [138,145]. Note that in this situation, the dentin graft particles are not just a scaffold for host bone growth, but they actually integrate with the bone to form a robust structure. Traditionally, graft materials have been assessed according to the degree of new bone growth they produce at different stages after grafting, with the volume of persisting graft being seen as a negative factor interfering with efficient reorganization of the site. In the case of dentin grafts, we propose that it is more meaningful to examine the percentage of ankylosis-like close bone-graft contacts, in addition to the host bone ingrowth. Unfortunately, this parameter is rarely assessed. A recent study by Artzi and colleagues recorded an average direct contact of dentin particles with bone of 69.1% at 6 months after grafting [135]. Using a qualitative assessment of bone graft contact, it was assessed that a dentin graft had good bone to graft contact in 85% of the cases 4 months after grafting, compared to just 40% of cases with a comparative bone graft [166].

### 5.3. Remodeling to Form Mechanically Optimized Bone Structure

Once a network of ankylosed new bone and dentin graft particles has formed, the resorption of the dentin proceeds only very slowly by external resorption replacement (Figure 3c). Essentially, the bone accepts the dentin as part of the normal bone structure and proceeds with resorption by the normal turnover mechanism of forming bone remodeling units, comprised of coordinated osteoclasts and osteoblasts, which are responsive to the mechanical stresses experienced [23]. Slow resorption is beneficial because it helps maintain the volume of the repair site. Deproteinized xenograft material has been shown to be excellent at maintaining volume, and recently high temperature allograft material for volume-enhancing purposes has been produced [3]. The disadvantage, however, is that the processing makes the material essentially non-resorbable, so that it eventually interferes with the establishment of a normal functional bone structure [40]. Dentin, however, achieves volume maintenance due to its natural structure and how it interacts with bone, and is then slowly resorbed without affecting bone structure.

### 5.4. Summary of Optimal Dentin-Derived Graft Use

**Figure 5:**
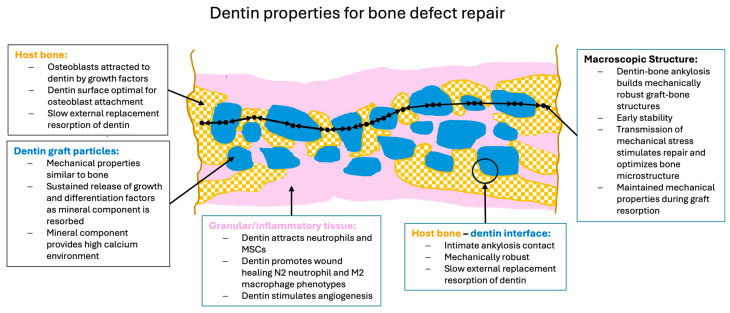
Summary of the key aspects whereby dentin properties positively influence bone repair.

Dentin-derived particulate graft material has many properties which indicate that it can be an excellent graft material (Figure 5). The particles contain organic and inorganic molecules which stimulate osteogenic repair by host cells. These molecules are protected in the inorganic matrix and released as the particles are slowly resorbed. The particles themselves have mechanical properties that match cortical bone and surface properties that encourage the adhesion and differentiation of both osteoblastic and osteoclastic cells. When the particles contact bone, they form an integrated mechanically stable unit and are then resorbed only slowly, via non-inflammatory, natural remodeling processes. Thus, the interaction of the dentin-derived particles with bone matches the natural healing phases.

Although even completely demineralized dentin ECM can be an effective graft material, the evidence does not support that the potentially higher earlier osteogenic factor release is a major gain in view of the loss of mechanical stability that accompanies the demineralization. Furthermore, exposure of the ECM by demineralization likely accelerates the degradation of osteogenic factors and the matrix compared to the sustained release from mineralized particles. The longer persistence of the graft material with dentin is not a problem because it integrates with the bone in a natural manner and is then slowly resorbed while maintaining mechanical strength. Optimal use thus requires the use of either fully demineralized or partially mineralized material. The greater early release of stimulatory factors with demineralization may, however, be an interesting way to tune the graft for use in cases where large defects are involved and part of the graft is far from the defect edges, thus requiring a greater osteoinductive stimulus in these areas. Short and limited demineralization of mineralized material just prior to use may be sufficient for this, without compromising the beneficial mechanical properties of the material.

## 6. Development of a Xenogeneic Dentin-Derived Material

### 6.1. Restrictions on Autogenous Dentin Graft Material Use

As described in the previous sections, autogenous dentin-derived bone graft material fulfills many of the criteria for an ideal bone graft material. It may be tuned to specific situations and has already been shown to be clinically effective in a variety of dental procedures, although larger studies with longer follow-ups are required to definitively compare it with other materials and establish its optimal use. Currently, however, a major restriction on the application of dentin-derived material is that sufficient healthy tooth material must be available to cover the requirements for the defects. Even for dental procedures where the amount of material required is generally lower than for orthopedic procedures, the amount of material available is often insufficient. It has been shown that the material can be extended with other graft materials, including autogenous bone [152] and xenogeneic deproteinized bone [133,151]. However, in clinical use, it would certainly be advantageous to have an off-the-shelf dentin-derived material which is available in sufficient amounts and can be applied to every patient without additional preparatory procedures. A potential alternative could be a xenogeneic material with retained organic material, but a literature search only revealed one study examining xenogeneic dentin material, and this was a deproteinized material that has not yet been tested in the clinic [167].

### 6.2. Development of a Xenogeneic Dentin-Derived Bone Graft Material

Our group has therefore developed Ivory Dentin Graft^TM^, a xenogeneic dentin-derived graft material with retained organic matrix, that can be produced in large amounts with consistent high quality, and which is safe and convenient for clinical use [166]. The production of the material is based on learnings from the use of chairside-prepared autogenous dentin and is designed to retain the beneficial properties of autogenous dentin while ensuring excellent biocompatibility and safety.

#### 6.2.1. Selection of Porcine Material

Porcine teeth were selected as the source for the material. Porcine tissue has been extensively used for clinical grafting procedures with a variety of tissues and so there is extensive experience concerning its use and compatibility [168,169]. Despite inevitable differences between porcine and human tissues, porcine tissue has generally been found to be the most similar to human tissue in comparison with other potential donor species. Due to the use of pigs as a standard research model, well-controlled tissues free from defined pathogens are abundantly available at relatively low cost. At the microstructural level, porcine dentin is more like human dentin than bovine, equine, or canine dentin, particularly in terms of tubular density [170]. In terms of inorganic nanocrystalline structure, it has been suggested that bovine dentin is the most similar to human dentin, although porcine dentin is in similar ranges and the functional consequences of such differences are unclear [171]. Demineralization of the porcine dentin matrix has been reported to require prolonged treatment compared to human dentin, suggesting that porcine dentin may be less readily resorbed than human dentin [172]. Proteomic analysis of the dentin organic ECM shows that while there are differences between porcine and human ECM, key evolutionary conserved molecules are similar in both materials, particularly with respect to bioactive molecules as shown by similar in vitro osteogenic activity [172,173]. In support of an anticipated similar clinical efficacy and safety of porcine graft material, there is a porcine bone graft material with retained organic matrix which has demonstrated efficacy and safety in dental bone-grafting procedures [42].

#### 6.2.2. Processing and Characterization of Porcine Dentin-Derived Graft Material

Porcine teeth were obtained from an isolated colony of pigs held under quality-controlled conditions, which had regular veterinary health controls and testing to ensure the absence of specific pathogens, and regular control and testing of feed and water to ensure the absence of any unwanted dietary contaminants. The teeth were then processed in an ISO 134865 [174] and ISO 9001 [175] certified facility with clean rooms. Processing of the material was similar to that of chairside autogenous dentin but with adapted incubation periods to ensure adequate cleaning, opening of tubules, and decontamination. The packaged material was then gamma radiation sterilized. The processing steps have been validated to eliminate even robust potential viral contamination according to ISO 22442 [176].

The porcine-dentin-derived graft material was extensively characterized to ensure that it met the required specifications in terms of reproducing the positive aspects of autogenous dentin while providing excellent safety and biocompatibility (Table 5).

Characterization of the material confirmed that it is a particulate with the size range 300–900 μm consisting of ca. 70% hydroxyapatite inorganic material and ca. 30% retained organic ECM material. Scanning electron microscopy confirmed irregularly shaped particles with open dentin tubules similarly to those seen with autogenic dentin. Porosity showed a high proportion of micropores consistent with the tubules for which ca. 80% were in the size range 0.7–1.5 μm. In addition, there were irregular coarse pores with a size range of 2–15 μm.

In comparison with bone-derived graft material, the dentin is denser and does not have the mesopore structure in the range of 40–60 μm associated with the trabecular structure. This may partially contribute to the slower resorption of the dentin material. X-ray diffraction confirmed the typical peak for hydroxyapatite with a peak width characteristic of nanocrystalline material which is more readily resorbed.

Microhardness measurements gave a mean value of Vickers hardness of 73 HV confirming the dense hardness of the particles compared to the range for human bone of 25–53 HV [177] and dentin of 48–70 HV [57,178]. The higher mean value may reflect some particles of enamel and also particles with low tubular content and perhaps some effect of processing.

Complete dissolution and elemental analysis of the material using inductively coupled plasma mass spectroscopy (ICP-MS) confirmed that no heavy metals or other metals were present at levels of toxicological concern. The calcium-to-phosphate ratio of the particles was determined to be in the range of 1.59–1.67. This indicates a slightly calcium deficient hydroxyapatite which indicates slightly higher solubility than stochiometric hydroxyapatite with a ratio of 1.67. Extracted amino acid analysis was consistent with a collagen-based ECM.

#### 6.2.3. Testing of Performance and Safety

The material has undergone extensive testing to establish both efficacy and safety and meets the stringent requirements of the EU MDR. There was no evidence of in vitro cytotoxicity of extracts (medium incubation for 72 h at 37 °C) in a standard mouse L929 fibroblast test using both cellular morphology and quantitative MTT assay as endpoints.

Implantation in a rabbit femoral condyle defect model in comparison to a porcine bone graft material with retained organic component demonstrated no intrinsically local adverse reactions, no local draining lymph node reaction, and no signs of systemic toxicity when examined 4 and 12 weeks after grafting. There was good host bone ingrowth at the implant sites and resorption of the porcine dentin graft material was much slower than the comparator porcine bone graft material.

Host bone regeneration and tolerability were superior to both deproteinated xenograft and sham controls in a canine mandibular two-wall defect model examined at 4, 12, and 24 weeks after grafting. At 24 weeks, there was greater persistence of graft material in the deproteinized xenograft bone material group than in the porcine dentin group, demonstrating that the dentin graft is more rapidly resorbed than the well-established xenograft material commonly used in dental clinical practice. Tolerability and initial inflammatory reaction were similar to the control deproteinized material.

In a clinically relevant porcine extraction socket and sub-periosteum pouch model, there was excellent defect repair already at 2.5 months after grafting. Grafted areas were solid, dense, and stable, and X-rays showed homogenous radio-opacity. Histology showed new bone growth in close apposition to the grafted particles.

A well-designed, randomized, parallel-group, semi-double-blinded clinical trial evaluated the safety, tolerability, and performance of the porcine dentin-derived material, in comparison with a clinically approved porcine bone material, in the indication of socket preservation and implant placement after mandibular premolar or molar extraction [148]. The graft material was well tolerated and had a similar adverse event and local tolerability profile to the comparator material. Titanium implant placement was successful in 95% of cases compared to 81.25% for the comparator.

**Figure 6:**
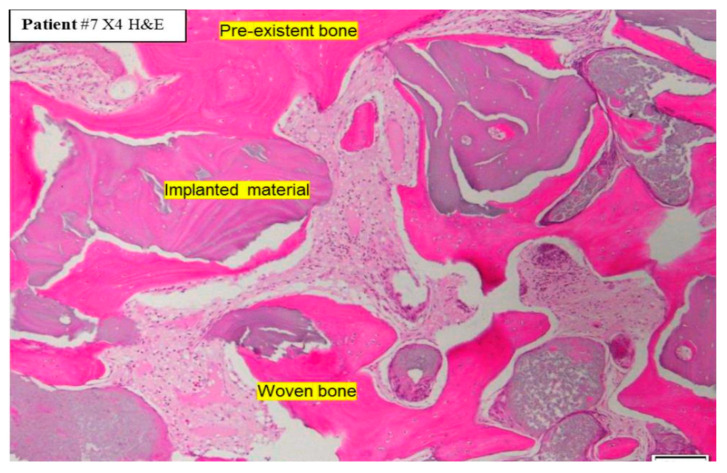
Representative histology of biopsy site at 4 months after grafting with Ivory Dentin Graft^TM^ for socket preservation [166]. Marker bar = 50 μm.

**Figure 7:**
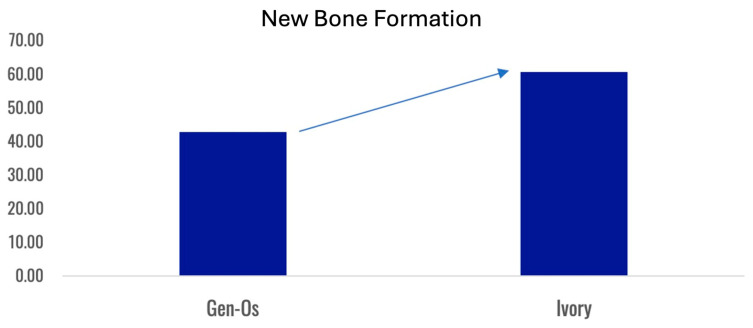
Mean percentage of new bone formation in graft site biopsies at 4 months after grafting for socket preservation. Ivory Dentin Graft^TM^ compared to the bone graft material Gen-Os [166].

**Figure 8:**
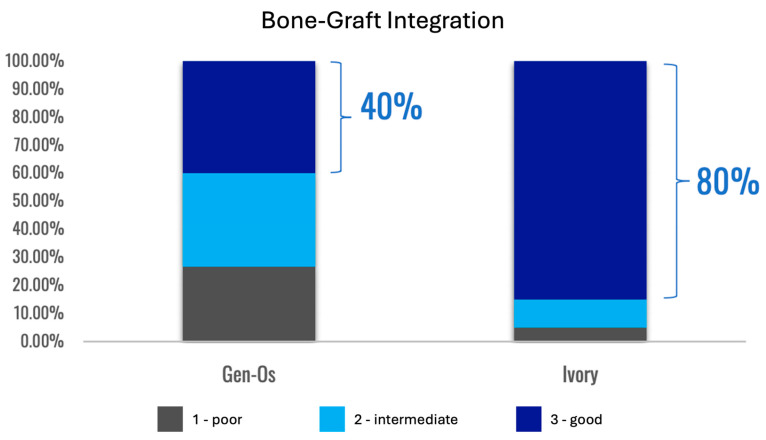
Bone-graft integration assessment of Ivory Dentin Graft^TM^ compared to the bone graft material Gen-Os, when used for socket preservation [166].

Histological analysis of biopsies taken at 4 months (Figure 6), at the time of implant placement, showed that the dentin graft material had statistically significantly more new bone formation than the bone graft comparator (Figure 7, 60.75% vs. 42.81%, *p* = 0.0084) and also better bone-graft integration scores (Figure 8, good integration 85% vs. 40% *p* = 0.0066). It should be noted that the comparator has a good clinical performance [42] and so showing superiority on these parameters is a very good performance. Implant placement at 4 months is also relatively early, demonstrating that the porcine dentin-derived graft material is able to generate a stable graft area relatively quickly, in line with the animal studies where there was a solid graft area already at 2.5 months.

**Figure 9:**
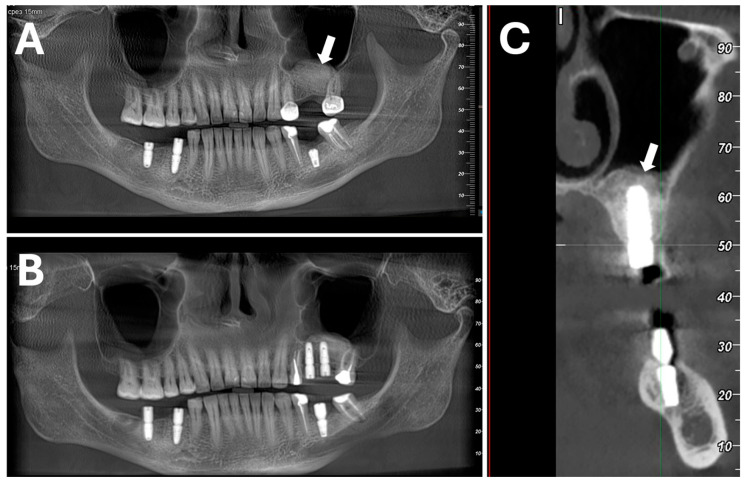
Use of Ivory Dentin Graft^TM^ for unilateral sinus augmentation. Status at 5 m when implants inserted. Radiographs show: (**A**) Left-side sinus wall augmentation (arrow); (**B**) implants inserted into augmented maxilla; (**C**) implant is well covered and supported by augmented bone (arrow). Unpublished case from Dr Sapoznikov of 72-year-old female.

Clinical experience is confirming that the xenogeneic dentin material is also very good for the whole range of indications for which autogenous dentin and the other graft materials are used. Apart from the socket preservation examined in the clinical study, there is now experience with alveolar ridge augmentation, cyst repair, guided bone regeneration, and sinus augmentation. Figure 9 illustrates the radiographic status in a representative case where the material was used for unilateral sinus augmentation resulting in good solid bone thickness increase, thus allowing dental implant placement at 5 months after grafting.

#### 6.2.4. Summary of New Porcine Dentin-Derived Graft Material

A new porcine dentin-derived graft material has been produced that shows similar performance to autogenous dentin and other available bone graft materials for dental bone defect repair. This allows the use of ‘off-the-shelf’ dentin graft material for cases where there is no, or insufficient, autogenous dentin material available. The material has a retained organic ECM component and shows similar performance to autogenic dentin. The material has been extensively characterized, is manufactured under strict quality control procedures, and is compliant with the strict EU Medical Device Regulation requirements. As for autogenic dentin, there is a need for more long-term follow-up data but based on the remodeling process of slow external replacement resorption no long-term problems would be anticipated. As summarized in Table 6 in a qualitative comparison this material compares favorably with the existing graft material options.

## 7. Conclusions and Future Perspectives

As summarized in this review, the dentin-derived graft material has properties that allow it to meet many of the criteria for an optimal graft material. Bone defect repair with dentin material proceeds differently to many other graft materials. Due to its density, it is more slowly resorbed than many bone-based materials and in this respect resembles high temperature-processed deproteinized bone, which is known for excellent volume-preserving characteristics. In contrast to the bone material, however, the dentin material is slowly resorbed as the bone and repair site remodels and therefore does not interfere with the ultimate long-term bone strength. The dentin material stimulates bone ingrowth but then forms intimate contacts with the bone which confers relatively early solidity and mechanical strength to the graft site.

These properties suggest that the dentin-derived material may be suited to a number of orthopedic uses where these properties could potentially be superior to existing materials. Availability of tooth-derived dentin graft material has been limited until now even for dental procedures, as the combination of defect and tooth extraction are required. With the availability of a high-quality dentin-derived graft material, we anticipate that there will be an expansion of dentin material use into the orthopedic area where it will be interesting to see how it will perform and how it can be adapted appropriately. For example, different particle size ranges and different degrees of demineralization may be better adapted for different particular uses. Also, the combination with other materials or substances such as autogenous bone, MSCs, platelet-rich fibrin, or various growth factors may be beneficial to extend the use in certain indications.

It is interesting to note that already shortly after the development of anesthesia allowed more invasive treatment of fractures, elephant ivory was found be an excellent material for bone repair [179]. Ivory, which is a form of dentin, was very biocompatible and exhibited no adverse reactions and when left in place was either integrated with the bone or partially resorbed. Ivory has also been used for hemiarthroplasty of the hip joint in Burma where several hundred patients were treated from the 1960s to 1995 [180]. The procedure was successful with cases of functional joints for up to 20 years after implantation. In cases that could be examined post-mortem, it was noted that there was close fusion of the ivory and bone and that in some areas the bone had incorporated into the ivory slowly replacing it. With the development of more modern methods and the stopping of elephant ivory use to preserve elephant populations, use of this material has discontinued. This early positive experience gives some further confidence that dentin-derived materials may have a place in orthopedics and ironically re-establish a practice from the very beginnings of modern orthopedics.

## Figures and Tables

**Table 1:** Properties of available bone graft materials.

Graft Material	Advantages	Disadvantages	Examples *
Autogenous bone	- Osteogenic - Osteoinductive - Osteoconductive - Good biocompatibility - Non-immunogenic - Low disease risk	- Second surgical site morbidity and complexity - Limited amount - Too rapid resorption in some cases - Low immediate strength - Variability	Mandibular bone Iliac crest
Allogenic bone	- Osteoinductive - Osteoconductive - Sufficient quantity - Ease of use	- Potential immunogenicity - Potential disease transmission - Too rapid resorption in some cases - Variability	FDBA, DBA
Xenogeneic bone deproteinized	- Osteoconductive - Maintains volume/slow resorption - Sufficient quantity - Ease of use	- Non-resorbable—persists, disrupting remodeling; only dental use - No organic component, thus lacks osteoinductive growth factors	Bio-Oss Cerabone
Xenogeneic bone retained ECM	- Osteoinductive - Osteoconductive - Sufficient quantity - Ease of use	- Potential immunogenicity - Potential disease transmission - Too rapid resorption in some cases	Osteobiol Gen Os
Alloplastic synthetics/ Bioceramics	- Osteoconductive - No disease risk - No immunogenicity risk - Tunable resorption - Good defect filling	- No osteoinductive potential via organic factors - Resorption often unpredictable	TCP HA Bioactive glass Calcium phosphates Calcium sulfate
Synthetic composites	- Osteoinductive - No disease risk	- High cost - Resorption unpredictable - Potential side effects	BMP-2/collagen PDGF-BB/TCP
Autogenous dentin	- Osteoinductive - Osteoconductive - Good biocompatibility - Early mechanical stability - Maintains volume/slow resorption - Non-immunogenic - Low disease risk	- Limited amount (for small defects only) - Dental procedures only - Not always available (tooth extraction required)	Smart Dentin Grinder Tooth Transformer Autobon VacuaSonic auto-FDT AutoBT

* Not exhaustive. Auto-FDT = autogenous fresh demineralized tooth; AutoBT = autogenous tooth bone graft; BMP-2 = bone morphogenic protein 2; DBA = demineralized bone allograft; FDBA = freeze-dried bone allograft; HA = hydroxyapatite; PDGF-BB = platelet-derived growth factor double-B subunits; TCP = tricalcium phosphate.

**Table 2:** Mechanical properties of dentin compared to bone and enamel.

Substance	Compressive Strength (MPa *)	Tensile Strength (MPa *)	Elasticity/Young’s Modulus (GPa *)
Dentin	250–350	21–160	11–19.7
Bone (cortical/compact)	88–200	124–174	3.9–18.9
Bone (trabecular/ spongeous **)	0.1–16	ND	0.05–0.5
Dentin (demineralized)	ND	30	0.02–0.21

* Stress in Pascals (N/m^2^); ** values dependent on density of specimen; ND = no data.

**Table 3:** Clinical use of autogenous dentin: review articles.

Clinical Applications	Authors and Year	Material Form	Mineralization	Aims and Conclusions
Alveolar ridge deficiency reconstruction	Ramanauskaite et al., 2019 [104]	particulate	all types	Systematic review examining the clinical efficacy of autogenous tooth material for alveolar ridge defect repair. Studies with limited patient numbers and short-term follow-up, support the potential of dentin material for alveolar ridge reconstruction.
Alveolar ridge augmentation, preservation, sinus augmentation, implant placement	Zhang et al., 2021 [105]	particulate	all types	Informal review comparing dentin graft material with other materials. Dentin has many advantages, including biocompatibility and osteoinductive properties.
Oral bone defects requiring augmentation (maxillary sinus lift, alveolar bone augmentation)	Li et al., 2022 [106]	particulate	all types ^1^	Meta-analysis using Cochrane and PRISMA methods of 7 RCTs comparing dentin with DBBM (Bio-Oss). Dentin significantly promoted bone regeneration and was not inferior to Bio-Oss.
Dental implant placement	Mahardawi et al., 2023 [107]	particulate	all types	Systematic review and meta-analysis using PRISMA methods, comparing implant stability for dentin compared to other materials in augmented sites. Dentin led to successful implant placement which, within the limited evidence base, was similar to autogenous bone and DBBM in terms of implant survival, ISQ, MBL, and peri-implant complications.
Sinus floor augmentation, alveolar ridge augmentation/preservation, cyst repair, implant placement	Murata et al., 2023 [14]	particulate/block	DDM	Informal review of animal models and clinical use of partial- and complete-DDM. Clinical data confirmed the efficacy of DDM for repair of a variety of dental bone defects.
Alveolar ridge preservation	Sánchez-Labrador et al., 2023 [108]	particulate	all types	Systematic review, using Cochrane and PRISMA methods, of the clinical outcomes with particulate dentin for alveolar ridge preservation procedures. Dentin was effective for ridge preservation with good volume maintenance, a high proportion of new bone growth and low complications. A need for more comparative studies with longer follow-up was identified.
Sinus floor augmentation, alveolar ridge augmentation/preservation, implant placement	Inchingolo et al., 2023 [101]	particulate	DDM	Systematic review using PRISMA methods to examine the preparation and clinical use of DDM. DDM was confirmed to be effective for bone repair for several dental procedures. Partial demineralization was considered better than complete demineralization to preserve growth factors in the material.
Socket preservation after tooth extraction	Madi et al., 2023 [109]	particulate	DDM	Systematic review using PRISMA methods to examine the clinical effects of different graft materials for socket preservation. DDM showed favorable results for socket preservation in terms of new bone formation, residual graft and ridge width.
Alveolar ridge augmentation	Mahardawi et al., 2023 [110]	block	MDM	Systematic review and meta-analysis using PRISMA methods to examine the efficacy of chairside MDM blocks compared to other materials for ridge augmentation. Chairside MDM blocks were successful for ridge augmentation. Evidence was weak due to the low number of comparative studies.
Socket preservation after tooth extraction	Feng et al., 2023 [111]	particulate	all types	Systematic review and meta-analysis using PRISMA methods to examine the clinical efficacy of dentin material for extraction socket ridge preservation compared to other graft materials or blood clot. Dentin material was more effective in maintaining ridge dimensions than blood clot healing, DBBM, or β-TCP and produced good new bone growth.
Alveolar ridge augmentation, sinus augmentation	Hashemi et al., 2024 [112]	particulate/block	all types	Systematic review using PRISMA methods to examine all systemic and meta-analysis reviews published up to August 2022. Autogenous tooth bone grafts appeared to be effective in oral defect reconstructions compared to Bio-Oss, autogenous bone blocks, or no-grafts. Additional long-term follow-up data are required, as are comparative studies, due to heterogenous methods and end points.
Sinus floor augmentation, alveolar ridge augmentation/preservation, cyst repair, implant placement	Khurshid et al., 2024 [113]	particulate	DDM	Review of the literature describing the potential of DDM for bone regeneration, including animal studies, clinical studies and reviews, and case reports. DDM was rated as an attractive option for bone regeneration and extracted socket preservation. Further studies are required to optimize the varied processing methods and for future therapeutic applications.
Alveolar ridge augmentation	Mahendra et al., 2024 [114]	particulate	all types	Systematic review using PRISMA methods to examine the effectiveness of dentin-derived alveolar bone graft for alveolar augmentation. Dentin-derived grafts resulted in better volume maintenance and higher new bone growth with low complications compared to controls. The influence of the degree of demineralization was unclear.
Sinus floor augmentation, alveolar ridge preservation, implant placement, guided bone regeneration	Sun et al., 2024 [115]	particulate	all types	Informal review of dentin-derived graft material composition, mechanisms of osteoinductivity, preparation, and clinical applications. Dentin provided a good alternative to autogenous bone in a variety of indications, but there is a need for longer term clinical efficacy data and more standardization in preparation procedures.
Sinus floor augmentation, alveolar ridge preservation, implant placement, bone defect repair	Olchowy et al., 2024 [116]	particulate	all types	Systematic review using PRISMA methods to examine the regenerative properties of dentin biomaterial, with a focus on standardized grinding protocols. Particularly in dental surgery, graft material derived from teeth is a promising alternative to bone autografts. Outcomes were positive across a wide range of processing methods.
Sinus floor augmentation, alveolar ridge augmentation/preservation, cyst repair, implant placement	Wysłouch et al., 2024 [117]	particulate	all types	High level narrative review explaining that tooth dentin graft material is a good viable alternative to autogenous bone.

DBBM = deproteinized bovine bone material; DDM = demineralized dentin material; ISQ = implant stability quotient; MBL = peri-implant marginal bone loss; MDM = mineralized dentin material; PRISMA = Preferred Reporting Items for Systemic Reviews and Meta-Analyses; RCT = randomized controlled trial. ^1^ The title of this review implies that it solely concerns demineralized material but is misleading because one of the major studies in the meta-analysis used MDM and this showed the greatest new bone formation of any of the studies.

**Table 4:** Clinical use of autogenous dentin: comparative studies.

Authors and Year	Study Design	Study Aims and Outcomes	Materials (Graft Site No.)	Results/Commentary
Sánchez-Labrador et al., 2020 [118]	Prospective, randomized split-mouth	Clinical parameters at 3 and 6 m after 3rd molar extraction with either MDM grafting or standard blood clot healing	MDM (15) vs. blood clot (15)	Compared to blood clot healing at 6 m, the MDM grafted sites had mean crestal bone height gain rather than loss, and greater bone density, both of which were statistically significant. Probing depths on the remaining 2nd molar were also significantly reduced compared to the control side.
Kuperschlag et al., 2020 [119]	Prospective, randomized, double arm, parallel group	Clinical and radiographic status at 3 and 12 m after impacted 3rd molar extraction with either MDM grafting or standard blood clot healing	MDM (13) vs. blood clot (11)	Compared to blood clot healing at 3 and 12 m, the MDM provided good bone growth with slow resorption, resulting in lower probing depth measurements.
Mazzucchi et al., 2022 [120]	Prospective, double arm, split-mouth	Clinical parameters at 6 m after third molar extraction with either MDM grafting or blood clot healing	MDM (10) vs. blood clot (10)	2nd molar pocket probing depths were decreased for both groups at 3 m and 6 m, with greater reduction for the dentin group, but only statistically significant at 3 m. Radiographic bone gain was greater for dentin at 6 m, but not statistically different. There was a trend for dentin graft to be better, but considerable within-group variability suggested the study was underpowered. The lack of membrane use may have contributed to the variability.
Hussain et al., 2023 [121]	Prospective, randomized, double arm, parallel group	Clinical, radiographic, and histological status at 4 m after grafting for ridge preservation after tooth extraction using either MDM or natural healing	MDM (14) vs. blood clot (15)	MDM maintained ridge height and width better than blood clot control, with greater new bone formation at 4 m after grafting.
Xu et al., 2023 [122]	Prospective, randomized, double arm, split-mouth	Clinical parameters of soft tissue repair at the alveolar ridge after ridge preservation following tooth extraction using either DDM or natural healing	DDM (22) vs. blood clot (22)	DDM (without membrane) better maintained gingival margin height at 30 days after grafting and showed more rapid soft tissue healing. At 3 days, there were more neutrophils in the dentin group associated with a quicker resolution of the initial inflammation.
López Sacristán et al., 2024 [123]	Prospective, randomized, double arm, split-mouth	Radiological and histological status at 2 and 4 m after grafting with MDM in comparison to natural healing (both with collagen membrane), for alveolar ridge preservation after tooth extraction	MDM (22) vs. blood clot (22)	MDM had less alveolar shrinkage than the controls. Not all parameters showed statistical significance, likely due to insufficient patient numbers. No inflammatory reaction to MDM was observed with intimate contact between the particles and newly grown bone. Dentin was confirmed to be an ideal slow resorption material for ridge preservation.
Gowda et al., 2023 [124]	Prospective, randomized, double arm, parallel group	Clinical and radiographic status at 4 m after alveolar ridge preservation grafting with either advanced PRF or PRF mixed with pDDM after tooth extraction	pDDM + PRF (8) vs. PRF (8)	pDDM mixed with PRF showed no significant radiographic reduction in alveolar dimensions at 4 m, whereas PRF-only treatment led to significant reduction. pDDM with PRF also had less reduction in clinical ridge dimensions compared to PRF.
Korsch and Peichl [125]	Retrospective, double-arm, case-series	Clinical parameters at 3 m after lateral ridge augmentation with tooth-shell method compared to bone-shell method	pDDM (38) vs. autogenous bone (41)	Block tooth with pDDM particles was as effective as autogenous bone block with autogenous bone particulate for alveolar ridge augmentation and implant placement. Radiographic evaluation, ISQ, and peri-implant probing depths were similar. A similar low complication rate was found for both materials.
Elraee et al., 2022 [126]	Prospective, randomized, double arm	Histomorphometric and clinical parameters at 6 m after upper central incisor horizontal ridge augmentation with either MDM block or bone block	MDM (21) vs. autogenous bone (21)	Both groups had uneventful healing and adequate clinical and radiographic ridge width gain but the MDM had statistically superior mean width at 6 m. Histology showed similar bone formation in both groups but slower resorption of MDM. The dentin integrated fully with the bone and was undergoing external replacement resorption. MDM was similar or even superior to autogenous bone in this context.
Pohl et al., 2021 [127]	Retrospective, case series, 5-arm	Clinical and radiographic assessment of soft tissue ingrowth behind socket shield tooth root between different graft materials	MDM (7) vs. PRF (7) vs. particulate autogenous bone (7) vs. autogenous cortical bone plate (7) vs. no graft (6)	MDM particles were more effective than autogenous bone particles for promoting bone growth to the exclusion of soft tissue. MDM was similarly effective to cortical bone plate. Slow resorption and high osteoinductivity were thought to be the critical parameters in this case.
Beldhi et al., 2024 [128]	Prospective, randomized, single-blinded, double arm	Radiographic status at 6 m after grafting with either MDM + PRF or demineralized freeze-dried allograft bone for ridge preservation after posterior tooth extraction	MDM + PRF (15) vs. DFDBA + PRF (15)	PRF-enhanced MDM showed less decrease in alveolar ridge dimensions at 6 m than PRF-enhanced allograft bone.
Oguić et al., 2023 [129]	Prospective, randomized, double arm	Radiographic and histomorphometric status at 4 m after post-extraction grafting in the aesthetic zone using either MDM or autogenous bone/DBBM mixture	MDM (20) vs. autogenous bone/DBBM—Cerabone (17)	There was no statistical difference at 4 m between the groups in either the radiographic ridge width or in the percentages of newly formed bone, residual graft material and soft tissue in the histology. MDM was therefore as good as the combination of osteogenic autogenous bone and non-resorbable xenogeneic material, in terms of maintaining volume and producing new bone growth in the very sensitive aesthetic zone.
Santos et al., 2021 [130]	Prospective, randomized, double arm, parallel group	Histomorphometry at 6 m post grafting, implant stability 2 m after placement, and clinical outcomes at 6, 12, and 18 m after placement were compared for post-extraction ridge preservation and delayed implant placement using either MDM or DBBM	MDM (26) vs. DBBM—Bio-Oss (26)	At 6 m, MDM showed more new bone growth and less graft material than DBBM. Primary implant stability was similar as was stability after 2 m. Peri-implant bleeding on probing had a similarly low incidence. Marginal bone loss was similarly low, as was the loss of keratinized gingival width. MDM had similar volume maintenance and implant stability to DBBM at up to 18 m follow-up.
Khalifah et al., 2023 [131]	Prospective, randomized, double arm	Clinical and radiographic status at 3, 6, and 12 m after immediate implant placement in anterior mandible using either MDM or DBBM.	MDM (28) vs. DBBM (28)	MDM and DBBM were statistically equivalent in terms of primary implant stability and implant stability at time of loading and 12 m. Plaque index, bleeding index, and probing depth were similarly equivalent. MDM, however, had lower implant stability at 3 and 6 m after loading, and greater bone loss prior to exposure. Although dentin can provide adequate stability in the aesthetic region with immediate implant placement, these preliminary results suggest that it is not as reliable as standard xenograft material due to a higher resorption rate.
Ouyang et al., 2024 [132]	Prospective, randomized, double arm	Radiographic status at 6 m and 2 years after alveolar ridge augmentation in orthodontic patients using either pDDM or DBBM	pDDM (20) vs. DBBM—Bio-Oss (20)	Both treatments achieved ridge augmentation that allowed for orthodontic tooth migration. At 6 m, the ridge width at 3 mm below apex was greater for pDDM than DBBM but 2-year dimensions were similar. Dentin was effective for augmentation but in the longer term was resorbed more than DBBM, although resulting in similar outcomes. pDDM had a milder post-operative response, suggestive of a relative anti-inflammatory effect compared to DBBM.
Minetti et al., 2019 [133]	Prospective, double arm case-series	Histomorphometry 4 m after tooth extraction socket repair using DDM compared to a 1:1 DDM/DBBM—(Bio-Oss)	DDM (3) vs. DDM/DBBM 1:1 (3)	Both DDM alone and combined with DBBM provided good ridge preservation at 4 m after grafting. Sites with dentin alone had more new bone growth and less retained graft material than those mixed with DBBM.
Wu et al., 2024 [134]	Prospective, randomized, three arm, parallel group	Clinical and radiographic status at 6 m after grafting with either MDM, TCP, or collagen sponge to improve bone healing after third molar retained root coronectomy	MDM (20) vs. TCP (19) vs. collagen sponge (19)	Although TCP facilitated bone healing compared to collagen sponge, the MDM was superior to TCP in terms of preventing retained root migration and rotation, and in bone embedding. It was also better in supporting the 2nd molar root. Thus, in a complex situation of a bone defect with retained tooth root and potential exposure of adjacent tooth root, the MDM rapidly established a mechanically robust site with good integrated new bone growth.

DBBM = deproteinized bovine bone material; DDM = demineralized dentin material; DFDBA = demineralized freeze-dried bone allograft; ISQ = implant stability quotient; m = months; mm = millimeters; MDM = mineralized dentin material; pDDM = partially demineralized dentin; PRF = platelet-rich fibrin; TCP = tricalcium phosphate.

**Table 5:** Properties of Ivory Dentin Graft^TM^.

Parameter	Aspect	Property/Specification
Raw material	Source	Health- and feed-controlled isolated pig colony
	Tissue	Porcine teeth
Material properties	Physical form	Irregular shaped particles, retaining dentin microstructure
	Particle size	300–900 μm
	Composition	ca. 70% hydroxyapatite and ca. 30% organic ECM (89% type I collagen, 1% proteoglycans, 10% others—non-collagenous proteins), partially degraded
	Vickers hardness	73 HV ± 14 HV
	Ca/P	1.59–1.67
	Microstructure	Porosity: 80% 0.7–1.5 μm tubules; 20% coarse pores 2–15 μm
Implant properties	Clinical bone growth	Superior new bone formation, bone-graft integration, and higher radiodensity than a porcine bone graft material with retained ECM at 4 months after mandibular premolar or molar tooth extraction in patients
	Preclinical bone growth	Superior to both deproteinated bone and sham treatment, in terms of bone regeneration and tolerability, in a canine mandibular two-wall defect model at 4, 12, and 26 weeks follow-up Good bone repair in extraction sockets and sub-periosteum pouches in a clinically relevant porcine mandibular defect model at 2.5 months after grafting
	Biocompatibility	In clinical use for socket preservation, local site reactions and adverse events following extraction socket grafting and implant placement were similar to that of a standard clinically established bone graft material In a rabbit femoral condyle defect model, no intrinsically local adverse reactions, no local draining lymph node reaction, and no signs of systemic toxicity In a canine mandibular two-wall defect model, tolerability and initial inflammatory reaction was similar to the control deproteinized bone material In a porcine extraction socket and sub-periosteum pouch model, only moderate inflammation consistent with normal healing was seen No in vitro cytotoxicity of extracts
	Resorption	In a canine two wall defect model, resorption up to 6.5 months was more rapid than for a deproteinated bovine bone material. In a rabbit condyle bone defect model, resorption assessed at 3 months was much slower than for a porcine bone material with retained organic ECM. Therefore, resorption is prolonged but not as long as for deproteinized xenograft bone graft material

Ca = calcium; ECM = extracellular matrix; HV = Vickers hardness; P = phosphate.

**Table 6:** Comparative qualitative analysis of bone graft properties.

Graft Type	Usability	Safety	Effectiveness	Overall Score
Dentin Autograft	++	+++	+++	8
Dentin Xenograft	+++	++	+++	8
Bone Autograft	+	+++	+++	7
Bone Allograft	++	+	++	5
Bone Xenograft	+++	+	++	6
Synthetic Graft	++	+++	+	6

+ = poor or moderate performance with complications; ++ = moderate performance or good with complications; +++ = good performance.

## Data Availability

All the data supporting reported results regarding publications cited in this review are available contacting the corresponding author upon request. Certain summarized data are the confidential proprietary property of Ivory Dentin Graft Ltd. These have been shared and reviewed by authorizing authorities but are not available upon request.
